# The Network of Global Corporate Control

**DOI:** 10.1371/journal.pone.0025995

**Published:** 2011-10-26

**Authors:** Stefania Vitali, James B. Glattfelder, Stefano Battiston

**Affiliations:** Chair of Systems Design, ETH Zurich, Zurich, Switzerland; Universidad Veracruzana, Mexico

## Abstract

The structure of the control network of transnational corporations affects global market competition and financial stability. So far, only small national samples were studied and there was no appropriate methodology to assess control globally. We present the first investigation of the architecture of the international ownership network, along with the computation of the control held by each global player. We find that transnational corporations form a giant bow-tie structure and that a large portion of control flows to a small tightly-knit core of financial institutions. This core can be seen as an economic “super-entity” that raises new important issues both for researchers and policy makers.

## Introduction

A common intuition among scholars and in the media sees the global economy as being dominated by a handful of powerful transnational corporations (TNCs). However, this has not been confirmed or rejected with explicit numbers. A quantitative investigation is not a trivial task because firms may exert control over other firms via a web of direct and indirect ownership relations which extends over many countries. Therefore, a complex network analysis [1] is needed in order to uncover the structure of control and its implications. Recently, economic networks have attracted growing attention [2], e.g., networks of trade [3], products [4], credit [5], [6], stock prices [7] and boards of directors [8], [9]. This literature has also analyzed ownership networks [10], [11], but has neglected the structure of control at a global level. Even the corporate governance literature has only studied small national business groups [12]. Certainly, it is intuitive that every large corporation has a pyramid of subsidiaries below and a number of shareholders above. However, economic theory does not offer models that predict how TNCs globally connect to each other. Three alternative hypotheses can be formulated. TNCs may remain isolated, cluster in separated coalitions, or form a giant connected component, possibly with a core-periphery structure. So far, this issue has remained unaddressed, notwithstanding its important implications for policy making. Indeed, mutual ownership relations among firms within the same sector can, in some cases, jeopardize market competition [13], [14]. Moreover, linkages among financial institutions have been recognized to have ambiguous effects on their financial fragility [15], [16]. Verifying to what extent these implications hold true in the global economy is *per se* an unexplored field of research and is beyond the scope of this article. However, a necessary precondition to such investigations is to uncover the worldwide structure of corporate control. This was never performed before and it is the aim of the present work.

## Methods

**Figure 1:**
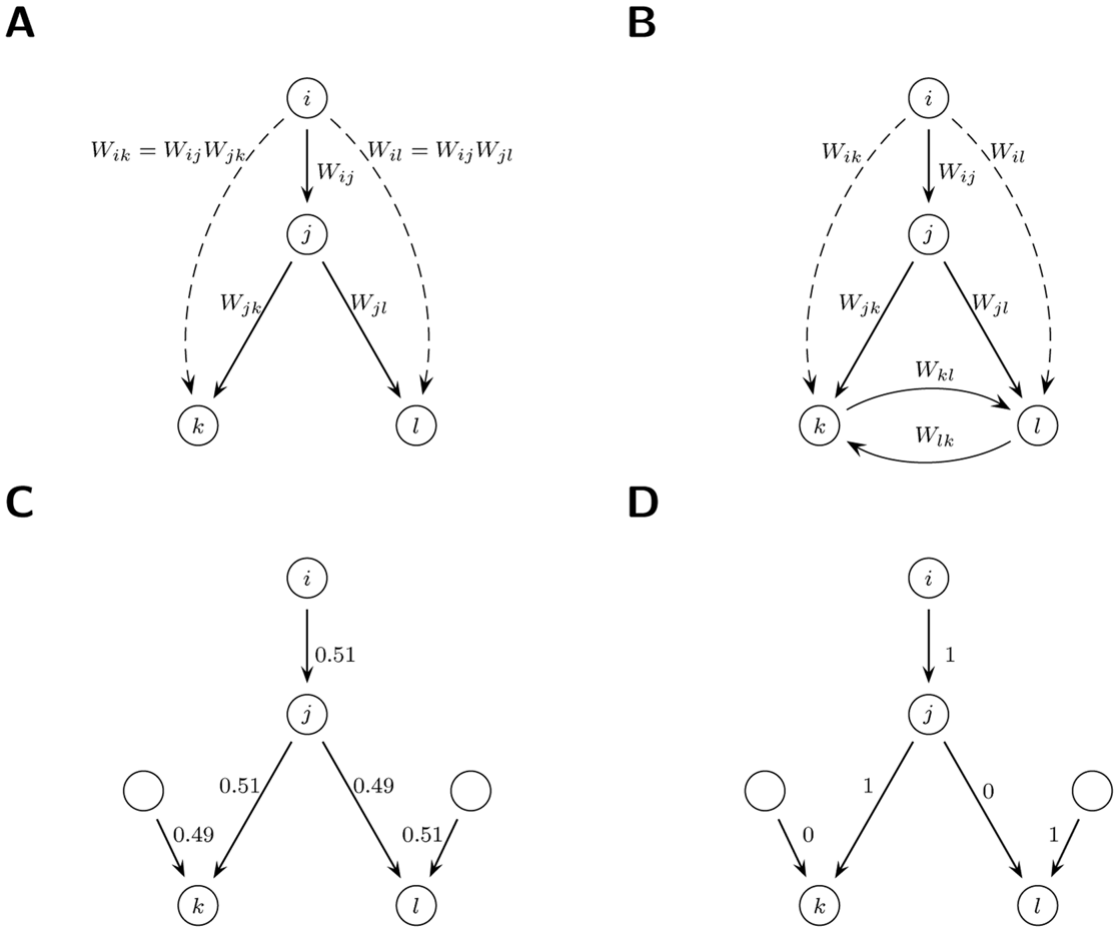
Ownership and Control. (**A&B**) Direct and indirect ownership. (A) Firm 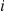 has 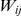 percent of direct ownership in firm 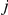. Through 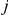, it has also an indirect ownership in 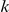 and 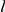. (B) With cycles one has to take into account the recursive paths, see Appendix S1, Section 3.1. (**C&D**) Threshold model. (C) Percentages of ownership are indicated along the links. (D) If a shareholder has ownership exceeding a threshold (e.g. 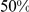), it has full control (100%) and the others have none (0%). More conservative model of control are also considered see Appendix S1, Section 3.1.

Ownership refers to a person or a firm owning another firm entirely or partially. Let 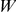 denote the ownership matrix, where the component 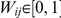 is the percentage of ownership that the owner (or *shareholder*) 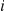 holds in firm 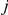. This corresponds to a directed weighted graph with firms represented as nodes and ownership ties as links. If, in turn, firm 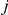 owns 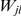 shares of firm 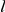, then firm 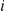 has an *indirect ownership* of firm 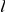 (Figure 1 A). In the simplest case, this amounts trivially to the product of the shares of direct ownership 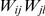. If we now consider the economic value 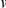 of firms (e.g., operating revenue in USD), an amount 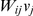 is associated to 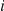 in the direct case, and 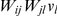 in the indirect case. This computation can be extended to a generic graph, with some important caveats [17], Appendix S1, Sections 3.1 and 3.2

Each shareholder has the right to a fraction of the firm revenue (dividend) and to a voice in the decision making process (e.g., voting rights at the shareholder meetings). Thus the larger the ownership share 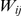 in a firm, the larger is the associated *control* over it, denoted as 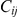. Intuitively, control corresponds to the chances of seeing one's own interest prevailing in the business strategy of the firm. Control 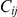 is usually computed from ownership 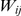 with a simple threshold rule: the majority shareholder has full control. In the example of Figure 1 C, D, this yields 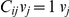 in the direct case and 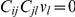 in the indirect case. As a robustness check, we tested also more conservative models where minorities keep some control (see Appendix S1, Section 3.1). In analogy to ownership, the extension to a generic graph is the notion of *network control*: 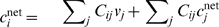. This sums up the value controlled by 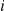 through its shares in 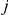, plus the value controlled indirectly via the network control of 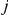. Thus, network control has the meaning of the total amount of economic value over which 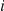 has an influence (e.g. 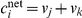 in Figure 1 D).

Because of indirect links, control flows upstream from many firms and can result in some shareholders becoming very powerful. However, especially in graphs with many cycles (see Figures 1 Band S4 in Appendix S1), the computation of 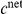, in the basic formulation detailed above, severely overestimates the control assigned to actors in two cases: firms that are part of cycles (or cross-shareholding structures), and shareholders that are upstream of these structures. An illustration of the problem on a simple network example, together with the details of the method are provided in Appendix S1, Sections 3.2–3.4. A partial solution for small networks was provided in [18]. Previous work on large control networks used a different network construction method and neglected this issue entirely [11], Appendix S1, Sections 2 and 3.5. In this paper, by building on [11], we develop a new methodology to overcome the problem of control overestimation, which can be employed to compute control in large networks.

## Results

We start from a list of 43060 TNCs identified according to the OECD definition, taken from a sample of about 30 million economic actors contained in the Orbis 2007 database (see Appendix S1, Section 2). We then apply a recursive search (Figure S1 and Section 2 in Appendix S1) which singles out, for the first time to our knowledge, the network of all the ownership pathways originating from and pointing to TNCs (Figure S2 in Appendix S1). The resulting TNC network includes 600508 nodes and 1006987 ownership ties.

Notice that this data set fundamentally differs from the ones analyzed in [11] (which considered only listed companies in separate countries and their direct shareholders). Here we are interested in the true global ownership network and many TNCs are not listed companies (see also Appendix S1, Section 2).

### Network Topology

**Figure 2:**
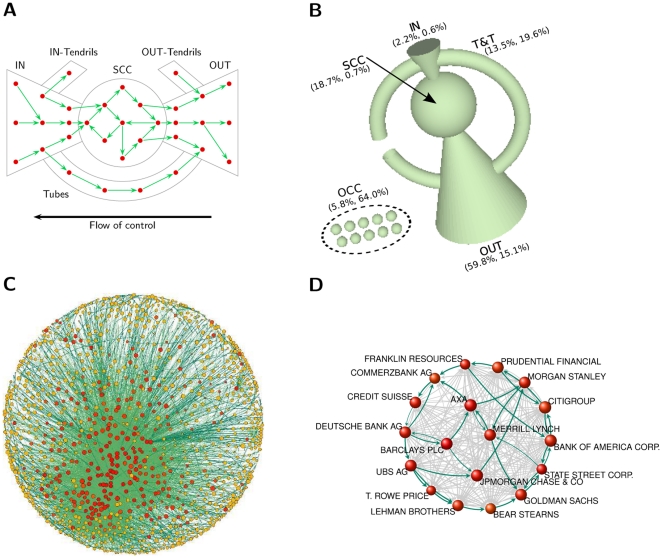
Network topology. (**A**) A bow-tie consists of in-section (IN), out-section (OUT), strongly connected component or core (SCC), and tubes and tendrils (T&T). (**B**) Bow-tie structure of the largest connected component (LCC) and other connected components (OCC). Each section volume scales logarithmically with the share of its TNCs operating revenue. In parenthesis, percentage of operating revenue and number of TNCs, cfr. Table 1. (**C**) SCC layout of the SCC (1318 nodes and 12191 links). Node size scales logarithmically with operation revenue, node color with network control (from yellow to red). Link color scales with weight. (**D**) Zoom on some major TNCs in the financial sector. Some cycles are highlighted.

The computation of control requires a prior analysis of the topology. In terms of connectivity, the network consists of many small connected components, but the largest one (3/4 of all nodes) contains all the top TNCs by economic value, accounting for 94.2% of the total TNC operating revenue (Table 1). Besides the usual network statistics (Figures S5 and S6 in Appendix S1), two topological properties are the most relevant to the focus of this work. The first is the abundance of cycles of length two (mutual cross-shareholdings) or greater (Figure S7 and Section 7 in Appendix S1), which are well studied motifs in corporate governance [19]. A generalization is a *strongly connected component* (SCC), i.e., a set of firms in which every member owns directly and/or indirectly shares in every other member. This kind of structures, so far observed only in small samples, has explanations such as anti-takeover strategies, reduction of transaction costs, risk sharing, increasing trust and groups of interest [20]. No matter its origin, however, it weakens market competition [13], [14]. The second characteristics is that the largest connect component contains only one dominant strongly connected component (1347 nodes). Thus, similar to the WWW, the TNC network has a *bow-tie* structure [21] (see Figure 2 A and Appendix S1, Section 6). Its peculiarity is that the strongly connected component, or *core*, is very small compared to the other sections of the bow-tie, and that the out-section is significantly larger than the in-section and the tubes and tendrils (Figure 2 B and Table 1). The core is also very densely connected, with members having, on average, ties to 20 other members (Figure 2 C, D). As a result, about 3/4 of the ownership of firms in the core remains in the hands of firms of the core itself. In other words, this is a tightly-knit group of corporations that cumulatively hold the majority share of each other.

**Table 1:** Bow-tie statistics.

	TNC (#)	SH (#)	PC (#)	OR (%)
LCC	15491	47819	399696	94.17
IN	282	5205	129	2.18
SCC	295	0	1023	18.68
OUT	6488	0	318073	59.85
T&T	8426	42614	80471	13.46
OCC	27569	29637	80296	5.83

Percentage of total TNC operating revenue (OR) and number (#) of nodes in the sections of the bow-tie (acronyms are in Figure 2). Economic actors types are: shareholders (SH), participated companies (PC).

Notice that the cross-country analysis of [11] found that only a few of the national ownership networks are bow-ties, and, importantly, for the Anglo-Saxon countries, the main strongly connected components are big compared to the network size.

### Concentration of Control

The topological analysis carried out so far does not consider the diverse economic value of firms. We thus compute the network control that economic actors (including TNCs) gain over the TNCs' value (operating revenue) and we address the question of how much this control is concentrated and who are the top control holders. See Figure S3 in Appendix S1 for the distribution of control and operating revenue.

**Figure 3:**
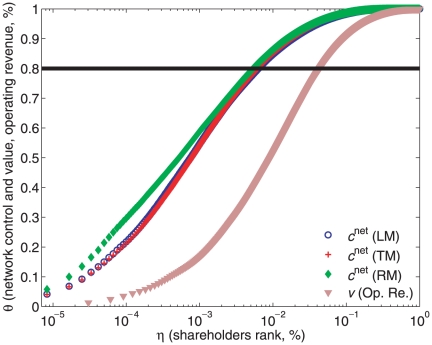
Concentration of network control and operating revenue. Economic actors (TNCs and shareholders) are sorted by descending importance, as given by 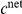. A data point located at (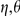) corresponds to a fraction 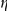 of top economic actors cumulatively holding the fraction 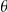 of network control, value or operating revenue. The different curves refer to network control computed with three models (LM, TM, RM), see Appendix S1, Section 3.1, and operating revenue. The horizontal line denotes a value of 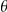 equal to 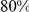. The level of concentration is determined by the 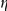 value of the intersection between each curve and the horizontal line. The scale is semi-log.

It should be noticed that, although scholars have long measured the concentration of wealth and income [22], there is no prior quantitative estimation for control. Constructing a Lorenz-like curve (Figure 3) allows one to identify the fraction 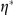 of top holders holding cumulatively 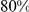 of the total network control. Thus, the smaller this fraction, the higher the concentration. In principle, one could expect inequality of control to be comparable to inequality of income across households and firms, since shares of most corporations are publicly accessible in stock markets. In contrast, we find that only 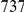 top holders accumulate 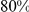 of the control over the value of all TNCs (see also the list of the top 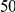 holders in Table S1 of Appendix S1). The corresponding level of concentration is 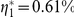, to be compared with 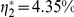 for operating revenue. Other sensible comparisons include: income distribution in developed countries with 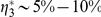 [22] and corporate revenue in Fortune1000 (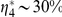 in 2009). This means that network control is much more unequally distributed than wealth. In particular, the top ranked actors hold a control ten times bigger than what could be expected based on their wealth. The results are robust with respect to the models used to estimate control, see Figure 3 and Tables S2 and S3 in Appendix S1.

## Discussion

The fact that control is highly concentrated in the hands of few top holders does not determine if and how they are interconnected. It is only by combining topology with control ranking that we obtain a full characterization of the structure of control. A first question we are now able to answer is where the top actors are located in the bow-tie. As the reader may by now suspect, powerful actors tend to belong to the core. In fact, the location of a TNC in the network does matter. For instance, a randomly chosen TNC in the core has about 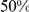 chance of also being among the top holders, compared to, e.g., 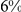 for the in-section (Table S4 in Appendix S1). A second question concerns what share of total control each component of the bow-tie holds. We find that, despite its small size, the core holds collectively a large fraction of the total network control. In detail, nearly 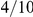 of the control over the economic value of TNCs in the world is held, via a complicated web of ownership relations, by a group of 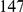 TNCs in the core, which has almost full control over itself. The top holders within the core can thus be thought of as an economic “super-entity” in the global network of corporations. A relevant additional fact at this point is that 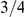 of the core are financial intermediaries. Figure 2 D shows a small subset of well-known financial players and their links, providing an idea of the level of entanglement of the entire core.

This remarkable finding raises at least two questions that are fundamental to the understanding of the functioning of the global economy. Firstly, what are the implication for global financial stability? It is known that financial institutions establish financial contracts, such as lending or credit derivatives, with several other institutions. This allows them to diversify risk, but, at the same time, it also exposes them to contagion [15]. Unfortunately, information on these contracts is usually not disclosed due to strategic reasons. However, in various countries, the existence of such financial ties is correlated with the existence of ownership relations [23]. Thus, in the hypothesis that the structure of the ownership network is a good proxy for that of the financial network, this implies that the global financial network is also very intricate. Recent works have shown that when a financial network is very densely connected it is prone to systemic risk [16], [24]. Indeed, while in good times the network is seemingly robust, in bad times firms go into distress simultaneously. This *knife-edge* property [25], [26] was witnessed during the recent financial turmoil.

Secondly, what are the implications for market competition? Since many TNCs in the core have overlapping domains of activity, the fact that they are connected by ownership relations could facilitate the formation of blocs, which would hamper market competition [14]. Remarkably, the existence of such a core in the global market was never documented before and thus, so far, no scientific study demonstrates or excludes that this international “super-entity” has ever acted as a bloc. However, some examples suggest that this is not an unlikely scenario. For instance, previous studies have shown how even small cross-shareholding structures, at a national level, can affect market competition in sectors such as airline, automobile and steel, as well as the financial one [13], [14]. At the same time, antitrust institutions around the world (e.g., the UK Office of Fair Trade) closely monitor complex ownership structures within their national borders. The fact that international data sets as well as methods to handle large networks became available only very recently, may explain how this finding could go unnoticed for so long.

Two issues are worth being addressed here. One may question the idea of putting together data of ownership across countries with diverse legal settings. However, previous empirical work shows that of all possible determinants affecting ownership relations in different countries (e.g., tax rules, level of corruption, institutional settings, etc.), only the level of investor protection is statistically relevant [27]. In any case, it is remarkable that our results on concentration are robust with respect to three very different models used to infer control from ownership. The second issue concerns the control that financial institutions effectively exert. According to some theoretical arguments, in general, financial institutions do not invest in equity shares in order to exert control. However, there is also empirical evidence of the opposite [23], Appendix S1, Section 8.1. Our results show that, globally, top holders are at least in the position to exert considerable control, either formally (e.g., voting in shareholder and board meetings) or via informal negotiations.

Beyond the relevance of these results for economics and policy making, our methodology can be applied to identify key nodes in any real-world network in which a scalar quantity (e.g., resources or energy) flows along directed weighted links. From an empirical point of view, a bow-tie structure with a very small and influential core is a new observation in the study of complex networks. We conjecture that it may be present in other types of networks where “rich-get-richer” mechanisms are at work (although a degree preferential-attachment [1] alone does not produce a bow-tie). However, the fact that the core is so densely connected could be seen as a generalization of the “rich-club phenomenon” with control in the role of degree [3], [28], Appendix S1, Section 8.2. These related open issues could be possibly understood by introducing control in a “fitness model” [29] of network evolution.

## Supporting Information

Appendix S1 Supporting material: Acronyms and abbreviations, Data and TNC Network Detection, Network Control, Degree and Strength Distribution Analysis, Connected Components Analysis, Bow-Tie Component Size, Strongly Connected Component Analysis, Network Control Concentration, Additional Tables.
(PDF)

## References

[1] Barabási A, Albert R (1999). Emergence of scaling in random networks.. Science.

[2] Schweitzer F, Fagiolo G, Sornette D, Vega-Redondo F, Vespignani A (2009). Economic networks: The new challenges.. Science.

[3] Fagiolo G, Reyes J, Schiavo S (2009). World-trade web: Topological properties, dynamics, and evolution.. Phys Rev E.

[4] Hidalgo C, Hausmann R (2009). The building blocks of economic complexity.. Proc Natl Acad Sci.

[5] Boss M, Elsinger H, Summer M, Thurner S (2004). Network topology of the interbank market.. Quant Financ.

[6] Iori G, De Masi G, Precup O, Gabbi G, Caldarelli G (2008). A network analysis of the Italian overnight money market.. J Econ Dyn Control.

[7] Bonanno G, Caldarelli G, Lillo F, Mantegna RN (2003). Topology of correlation-based minimal spanning trees in real and model markets.. Phys Rev E.

[8] Strogatz S (2001). Exploring complex networks.. Nature.

[9] Battiston S, Catanzaro M (2004). Statistical properties of corporate board and director networks.. Eur Phys J B.

[10] Kogut B, Walker G (2001). The small world of germany and the durability of national networks.. Amer Sociol Rev.

[11] Glattfelder JB, Battiston S (2009). Backbone of complex networks of corporations: The flow of control.. Phys Rev E.

[12] Granovetter M (1995).

[13] O'Brien D, Salop S (1999). Competitive Effects of Partial Ownership: Financial Interest and Corporate Control.. Antitrust Law J.

[14] Gilo D, Moshe Y, Spiegel Y (2006). Partial cross ownership and tacit collusion.. RAND J Econ.

[15] Allen F, Gale D (2000). Financial contagion.. J Polit Econ.

[16] Stiglitz JE (2010). Risk and global economic architecture: Why full financial integration may be undesirable.. http://www.nber.org/papers/w15718.

[17] Brioschi F, Buzzacchi L, Colombo M (1989). Risk capital financing and the separation of ownership and control in business groups.. J Bank Financ.

[18] Baldone S, Brioschi F, Paleari S (1998). Ownership Measures Among Firms Connected by Cross-Shareholdings and a Further Analogy with Input-Output Theory.. 4th JAFEE International Conference on Investment and Derivatives.

[19] Dietzenbacher E, Temurshoev U (2008). Ownership relations in the presence of cross-shareholding.. J Econ.

[20] Williamson O (1975). Markets and hierarchies, analysis and antitrust implications: a study in the economics of internal organization..

[21] Broder A, Kumar R, Maghoul F, Raghavan P, Rajagopalan S (2000). Graph structure in the Web.. Comput Netw.

[22] Atkinson A, Bourguignon F (2000). Handbook of income distribution.. Elsevier.

[23] Santos J, Rumble A (2006). The American keiretsu and universal banks: Investing, voting and sitting on nonfinancials' corporate boards.. J Finan Econ.

[24] Battiston S, Delli Gatti D, Gallegati M, Greenwald B, Stiglitz J (2009). Liaisons dangereuses: Increasing connectivity, risk sharing and systemic risk..

[25] Alesandri P, Haldane A (2009). Banking on the state.. http://www.bankofengland.co.uk/publications/speeches/2009/speech409.pdf.

[26] May R, Levin S, Sugihara G (2008). Ecology for bankers.. Nature.

[27] La Porta R, de Silanes FL, Shleifer A (1999). Corporate ownership around the world.. J Finance.

[28] Colizza V, Flammini A, Serrano M, Vespignani A (2006). Detecting rich-club ordering in complex networks.. Nat Phy.

[29] Garlaschelli D, Capocci A, Caldarelli G (2007). Self-organized network evolution coupled to extremal dynamics.. Nat Phys.

