# Supplementary material for: The Network of Global Corporate Control
Source: PLoS One. 2011 Oct 26;6(10):e25995. doi: 10.1371/journal.pone.0025995 (PMC3202517; doi:10.1371/journal.pone.0025995)
Supplement: Appendix S1 — Supporting material: Acronyms and abbreviations, Data and TNC Network Detection, Network Control, Degree and Strength Distribution Analysis, Connected Components Analysis, Bow-Tie Component Size, Strongly Connected Component Analysis, Network Control Concentration, Additional Tables. (PDF) [file pone.0025995.s001.pdf]

Supporting Information: The Network of Global Corporate Control

Stefania Vitali, James B. Glattfelder and Stefano Battiston
Chair of Systems Design, ETH Zurich, Kreuzplatz 5, 8032 Zurich, Switzerland

Contents

1 Acronyms and Abbreviations **2**

2 Data and TNC Network Detection **3**

3 Network Control **5**
3.1 The Existing Methodology 5
3.2 The Algorithm: Computing Control While Remedying the Problems 7
3.3 Proving the BFS Methodology Corrects for Cycles 8
3.4 An Illustrated Example 10
3.5 Relations To Previous Work 11

4 Degree and Strength Distribution Analysis **12**

5 Connected Component Analysis **13**

6 Bow-Tie Component Sizes **14**

7 Strongly Connected Component Analysis **15**

8 Network Control Concentration **16**
8.1 Control of Financial Institutions 16
8.2 Relation to the Rich Club Phenomenon 16
8.3 Top Control-Holders Ranking 16

9 Additional Tables **18**

1 Acronyms and Abbreviations

The list of acronyms and abbreviations used in the main text and this Supporting Online Material:

BFS: breadth-first search (search algorithm)
CC: (weakly) connected component
FS: financial sector
IN: in-section of a bow-tie
LCC: largest CC
LM: linear model (for estimating control from ownership; see also RM and TM)
NACE: (industry standard classification system)
OCC: other connected components (everything outside the LCC)
OECD: Organization for Economic Co-operation and Development
OR: operating revenue
OUT: out-section of a bow-tie
PC: participated company
RM: relative model (for estimating control from ownership; see also LM and TM)
SCC: strongly connected component (in the main text, this is synonymous with the core of the bow-tie in the LCC)
SH: shareholder (economic actors holding shares in TNCs)
TCH: top control-holder (list of TNCs and SHs that together hold 80% of the network control)
TM: threshold model (for estimating control from ownership; see also LM and RM)
TNC: transnational corporation (OECD definition)
T&T: tubes and tendrils (sections in a bow-tie that either connect IN and OUT, are outgoing from IN, or ingoing to OUT, respectively)

2 Data and TNC Network Detection

The Orbis 2007 marketing database[1] comprises about 37 million economic actors, both physical persons and firms located in 194 countries, and roughly 13 million directed and weighted ownership links (equity relations). Among many others, information on the industrial classification, geographical position and operating revenue of the actors are provided. This data set is intended to track control relationships rather than patrimonial relationships. Whenever available, the percentage of ownership refers to shares associated with voting rights.

The definition of TNCs given by the OECD [1] states that they

> *[...] comprise companies and other entities established in more than one country and so linked that they may coordinate their operations in various ways, while one or more of these entities may be able to exercise a significant influence over the activities of others, their degree of autonomy within the enterprise may vary widely from one multinational enterprise to another. Ownership may be private, state or mixed.*

Accordingly, we select those companies which hold at least 10% of shares in companies located in more than one country. However, many subsidiaries of large TNCs fulfill themselves this definition of TNCs (e.g. The Coca-Cola Company owns Coca-Cola Hellenic Bottling Company which in turn owns Coca-Cola Beverages Austria). Since for each multinational group we are interested in retaining only one representative, we exclude from the selection the companies for which the so-called ultimate owner (i.e., the owner with the highest share at each degree of ownership upstream of a company[1]) is quoted in a the stock market. In substitution, we add the quoted ultimate owner to the list (if not already included). In the example above, this procedure identifies only the Coca-Cola Company as a TNC. Overall we obtain a list of 43060 TNCs located in 116 different countries, with 5675 TNCs quoted in stock markets.

Starting from the list of TNCs, we explore recursively the neighborhood of companies in the whole database. First, we proceed downstream of the TNCs (see Fig. S1) with a breadth-first search (BFS) and we identify all companies participated directly and indirectly by the TNCs. We then proceed in a similar way upstream identifying all direct and indirect shareholders of the TNCs. The resulting network can be divided into three classes of nodes, TNC, SH and PC, as shown in Fig. S2. The TNC network constructed in this way consists of 600508 economic entities and 1006987 corporate relations. Notice that it may be possible to reach a PC from several TNCs, or to reach a TNC from several SHs. In other words, paths proceeding downstream or upstream of the TNCs may overlap, giving rise to CCs of various sizes.

It is worthwhile to distinguish the data set constructed here from the one analysed in [5], which was not obtained using a recursive search, but with the simple method of collecting only listed companies and their direct shareholders. This method neglects all indirect paths involving non-listed companies, so that the true ownership network was only approximated. Moreover, 48 countries were analysed separately, ignoring all cross-country links, an approach which inevitably leaves out entirely the global structure of ownership. The aim there was to construct disjoint national stock market networks, from which the backbones were extracted and analyzed. Here, however, we focus on the entire global topology.

[1]URL: `http://www.bvdep.com/en/ORBIS`.

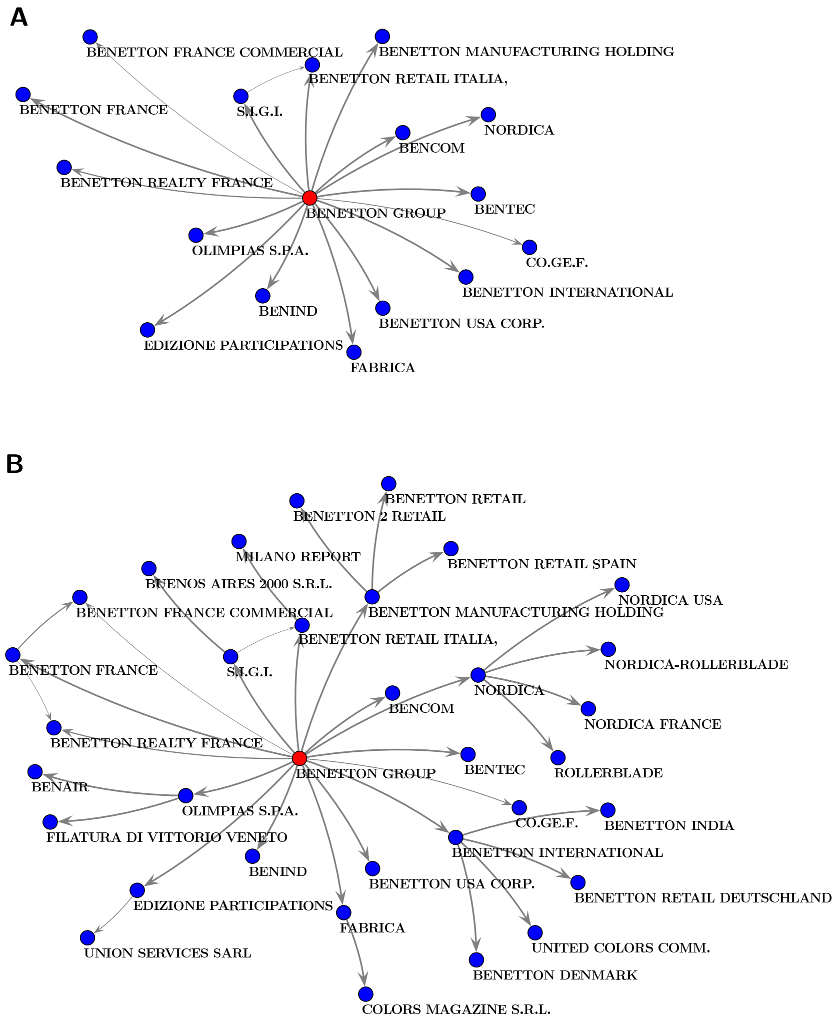

Figure S1. Illustration of the first two steps in the recursive exploration downstream of a TNC. Starting from "Benetton Group" the BFS explores all the direct neighbors (**A**), and then the neighbors' neighbors (**B**).

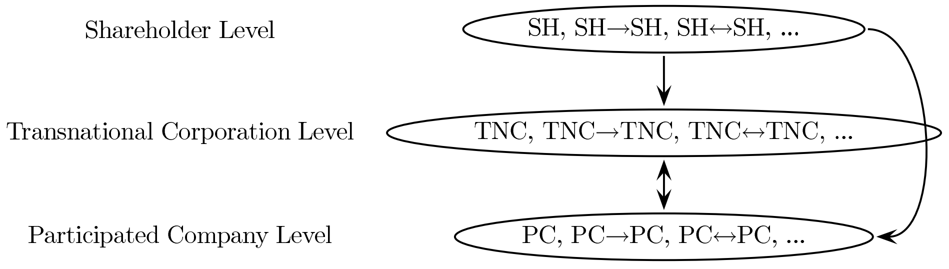

Figure S2. General structure of the TNC network. Three types of economic actors appear: 77456 SHs, 43060 TNCs and 479992 PCs. The network contains in total 600508 nodes, and 1006987 links. Links are mainly from the TNCs to the PCs and amongst the PCs themselves.

3 Network Control

In this section, we first recapitulate in detail the existing method for computing the value or control in a network. In a second step, we highlight two problems that plague this approach, especially in networks with bow-tie topology (see main text, Sec. Network Topology). The first is that the control assigned to firms that are part of cross-shareholding structures is overestimated. The second is a similar overestimation of the control of the shareholders who are themselves not owned by others. These two problems require independent solutions. In particular, the second problem was never raised before in the literature. We provide a novel algorithm that, for the first time, solves both problems and allows the computation of control also for large networks. This method represents a fundamental improvement to previous works, including our own one [5], as explained below in details. Finally, we illustrate the problem and the corrections introduced by the algorithm using a representative example of a small bow-tie network.

3.1 The Existing Methodology

While ownership is an objective quantity given by the percentage of shares owned in a company, control, reflected in voting rights, can only be estimated using a model. There are two steps involved in the derivation of the notion of control we use in this work. Firstly, direct control is estimated from the direct ownership relations. Network control is then computed on the basis of direct control considering all paths in the network.

For the computation of the direct control, we use three models: the linear model, applying the one-share-one-vote rule [2, 3], the threshold model [4] and the relative control model [5]. In the main part of the text, we denote these three models as LM, TM and RM, respectively. According to the LM, there is no deviation between ownership and control, thus the direct control matrix coincides with the ownership matrix, $L_{ij} = W_{ij}$. In the TM, full control over a company is assigned to the actor holding a number of shares higher than a predefined threshold (50% in our case), while the other holders are assigned zero control. The control matrix for the threshold model is denoted as T_{ij}. Finally, the RM assigns control based on the relative fraction of ownership shares that each shareholder has (using a Herfindhal-like concentration index). The control matrix is defined as $R_{ij} := W_{ij}^2/(\sum_{l=1}^{k_j^{in}} W_{lj}^2)$. In particular, the RM assigns high control to a shareholder with a small share in absolute terms, if this share is significantly bigger than the shares of all the other shareholders. For each of these three control matrices, network control is computed with the same procedure. In the main text we use the TM as our main measure, and compare all the results with the LM and the RM. It should be stressed that the global findings are insensitive to the chosen model of direct control.

As explained in the main text, the value of the portfolio of firms owned directly by i should be computed taking into account the value of the firms owned by the firms in the portfolio and so on. Thus, the network portfolio value p_i^{net} consists of the value gained indirectly plus the value of the direct portfolio: $p_i^{\text{net}} = \sum_j W_{ij} v_j + \sum_j W_{ij} p_j^{\text{net}}$. The vector v represents the intrinsic value of the firms (e.g., operating revenue, total assets or market capitalization). Here we use operating revenue, because it is readily available for the economic actors under investigation and it is comparable across sectors (this is not true for total assets). In analogy to the definition above, we introduce the *network control (value)* [5]. This quantity measures the value controlled by a shareholder taking into account the network of firms in which it has direct or indirect shares. In matrix notation,

$$c^{\text{net}} = \mathcal{C} c^{\text{net}} + \mathcal{C} v, \tag{1}$$

where $\mathcal{C} \in \{L, T, R\}$ is one of the three direct control matrices. The solution to Eq. (1) is given by

$$c^{\text{net}} = (I - \mathcal{C})^{-1} \mathcal{C} v =: \tilde{\mathcal{C}} v. \tag{2}$$

For the matrix $(I-\mathcal{C})$ to be non-negative and non-singular, a sufficient condition is that the Frobenius root of $\mathcal{C}$ is smaller than one, $\lambda(\mathcal{C}) < 1$. This is ensured by the following requirement: in each strongly connected component $\mathcal{S}$ there exists at least one node j such that $\sum_{i\in\mathcal{S}} \mathcal{C}_{ij} < 1$. This means that there exists no subset of k firms ($k = 1, \ldots, n$) that are entirely controlled by the k firms themselves, a condition which is always fulfilled.

By taking the series expansion of $(I-\mathcal{C})^{-1}$, it can be proven that: $\mathcal{C}(I-\mathcal{C})^{-1} = (I-\mathcal{C})^{-1}\mathcal{C}$. As a consequence, $\tilde{\mathcal{C}}$ in Eq. (2) coincides with the solution to the equation

$$\tilde{\mathcal{C}}_{ij} = \mathcal{C}_{ij} + \sum_k \tilde{\mathcal{C}}_{ik}\mathcal{C}_{kj}. \tag{3}$$

This corresponds to the definition of integrated ownership given in [6]. Hence, as in [5], we can interpret c^{net} as the value of control an economic actor gains from all its direct and indirect paths in the network.

Notice that Eq. (1) is related to the notion of eigenvector centrality used to investigate power and influence both in social and economic networks [7,8]. There is also an additional interpretation of network control in terms a physical system in which a quantity is flowing along the links of the network [5]. In this picture, nodes associated with a value v_j produce v_j units of the quantity at time $t = 1$. The weight of a link ij, given by the adjacency matrix entry A_{ij}, determines the fraction of v_j that flows through it. Then the *inflow*, i.e. the flow ϕ_i entering the node i from each node j at time t is the fraction A_{ij} of the quantity produced by j plus the same fraction of the inflow of j:

$$\phi_i(t+1) = \sum_j A_{ij}\phi_i(t) + \sum_j A_{ij}v_j, \tag{4}$$

In matrix notation, at the steady state, this yields

$$\phi = A\phi + Av, \tag{5}$$

which is formally identical to Eq. (1). Thus if v corresponds to an intrinsic economic value of the nodes, then the network control corresponds to the inflow of control over this value. The network portfolio value of a node is determined by the total inflow of value entering the node.

Next to network control, a related quantity is the so-called *network value*

$$v^{\text{net}} = \mathcal{C}v^{\text{net}} + v, \tag{6}$$

which is akin to a Hubbell index centrality measure [9]. This measure is well-established in the literature [6]. The solution is $v^{\text{net}} = (I-\mathcal{C})^{-1}v$. By noting that

$$\mathcal{C}v^{\text{net}} = \mathcal{C}(I-\mathcal{C})^{-1}v = \tilde{\mathcal{C}}v, \tag{7}$$

we find

$$v^{\text{net}} = \tilde{\mathcal{C}}v + v = c^{\text{net}} + v. \tag{8}$$

In other words, the network value of an economic actor is given by its intrinsic value plus the value gained from network control. It is an estimate of the overall value a corporation has in an ownership network. Notice that network value and network control of a company can differ considerably. As an example, Wall Mart is in top rank by operating revenue but it has no equity shares in other TNCs and thus its network control is zero. In contrast, a small firm can acquire enormous network control via shares in corporations with large operating revenue.

From Eq. (7), where $c^{\text{net}} = \tilde{\mathcal{C}}v = \mathcal{C}v^{\text{net}}$, network control can either be understood as the value of control gained from the intrinsic value reachable by all direct and indirect paths or the value of control given by the network value of directly controlled firms.

3.2 The Algorithm: Computing Control While Remedying the Problems

Unfortunately, the equations defining network control and network value suffer from three drawbacks. Firstly, the computation overestimates control when there are cycles in the network (for example in an SCC[2]), i.e., when the number of inter-firm cross-shareholdings grows [10]. Secondly, as we have discovered, it also leads to paradoxical situations. Consider for instance an SCC that is reachable from a single root-node r that owns an arbitrarily small share in one of the firms in the SCC. The above definition assigns to such a node the sum of the intrinsic value of all the nodes in the SCC. This is obviously not a correct estimate of the control of the node r. These two issues are best understood in the flow analogy. Indeed, in a dense SCC control flows through the nodes many times. The smaller the incoming links from the IN are the longer it takes until the flow stops, as, in the steady state, everything ultimately flows to and accumulates in the root-nodes. However, since control corresponds to the total inflow over an infinite time this exaggerates the control of the nodes in the SCC and all the control ultimately flows to the root-nodes. Thirdly, for large networks, the computation of the inverse matrix can be intractable. Here, for the first time, we overcome the aforementioned problems and propose a new methodology that consists of applying an algorithm to compute network control by treating different components of the network separately.

We first illustrate the algorithm for the computation of v^{net}. Then $c^{\text{net}} = v^{\text{net}} - v$. In order to calculate the network value for any specific node i, we extract the whole subnetwork that is downstream of a node i, including i. For this purpose, a breadth-first-search (BFS) returns the set of all nodes reachable from i, going in the direction of the links. Then, all the links among these nodes are obtained from the control matrix of the entire network, except for the links pointing to i which are removed. This ensures that there are no cycles involving i present in the subnetwork. Let $B(i)$ denote the adjacency matrix of such a subnetwork, including i, extracted from the control matrix $\mathcal{C} = (L, T, R)$. Without loss of generality, we can relabel the nodes so that $i = 1$. Since node 1 has now no incoming links, we can decompose $B = B(1)$ as follows:

$$B = \left(\begin{array}{c|c} 0 & d \\ \hline \vec{0} & B^{\text{sub}} \end{array} \right), \tag{9}$$

where d is the row-vector of all links originating from node 1, and B^{sub} is associated with the subgraph of the nodes downstream of i. The value of these nodes is given by the column-vector v^{sub}. By replacing the the matrix B in the expression $v^{\text{net}} = \tilde{\mathcal{C}} v + v = \mathcal{C}(I - \mathcal{C})^{-1} v + v$ and taking the first component we obtain:

$$\begin{aligned} v^{\text{net}}(1) &= \left[B(I-B)^{-1}v\right]_1 + v_1 \\ &= d(I^{\text{sub}} - B^{\text{sub}})^{-1} v^{\text{sub}} + v_1 =: \tilde{d} \cdot v^{\text{sub}} + v_1, \end{aligned} \tag{10}$$

where now $c^{\text{net}}(1) := \tilde{d} \cdot v^{\text{sub}} = d(I^{\text{sub}} - B^{\text{sub}})^{-1} v^{\text{sub}}$.

Notice that if node i has zero in-degree, this procedure yields the same result as the previous formula: $\tilde{B}_{(i,*)} = (0, \tilde{d}) = \tilde{\mathcal{C}}_{(i,*)}$. The notation $A_{(i,*)}$ for a matrix is understood as taking its i-th row. In the next section it is shown that our calculation is in fact equivalent to the correction proposed by [10] to address the problems of the overestimation of network value in the case of ownership due to the presence of cycles.

However, both methods still suffer from the problem of root nodes accumulating all the control. This issue was previously overlooked because the cases analysed did not have a bow-tie structure and because the focus was not on the empirical analysis of control. To solve this issue, we adjust our algorithm to pay special attention to the IN-nodes of an SCC. We partition the bow-tie associated with this SCC into its components: the IN (to which we also add the T&T), the SCC itself, and the OUT. Then, we proceed in multiple steps to compute the network value for all parts in sequence. In this way, the control flows

[2]For more information see SI Sec. 7.

from the OUT, via the SCC to the IN. Finally, the network control is computed from the network value as $c^{\text{net}} = v^{\text{net}} - v$. In detail, our algorithm works as follows:

1. OUT: Compute the network value $v^{\text{net}}(i)$ for all the nodes in the OUT using Eq. (10).

2. OUT $\rightarrow$ SCC: Identify the subset $\mathcal{S}1$ of nodes in the SCC pointing to nodes in the OUT, the latter subset denoted as $\mathcal{O}$. To account for the control entering the SCC from the OUT, compute the network value of these selected nodes by applying $v^{\text{net}}(s) = \sum_o \mathcal{C}_{so} v^{\text{net}}(o) + v_s$ to them. This is an adaptation of Eq. 8, where s and o are labels of nodes in $\mathcal{S}1$ and $\mathcal{O}$, respectively. Note that we only needed to consider the direct links for this. This computation is also equivalent to applying Eq. (10), which considers the downstream subnetworks of $\mathcal{S}1$, i.e., the whole OUT.

3. SCC: Employ Eq. (10) to the SCC-nodes restricting the BFS to retrieve only nodes in the SCC itself. Note that for those SCC-nodes that were already considered in step 2, their network value is now taken as the intrinsic value in the computation. This means one first needs to assign $v_i \mapsto v^{\text{net}}(i) + v_i$.

4. SCC $\rightarrow$ IN: In this step we solve the problem of the root-nodes acquiring an exaggerated fraction of the network value. For the subset of IN-nodes $\mathcal{I}$ directly connected to some SCC-nodes $\mathcal{S}2$, we again apply $v^{\text{net}}(i) = \sum_s \mathcal{C}_{is} v^{\text{net}}(s) + v_i$, where i and s are labels of nodes in $\mathcal{I}$ and $\mathcal{S}2$, respectively. However, note that due to the cycles present in the SCC, this computation is not equivalent to Eq. (10). In this way only the share of network value over the SCC which is not owned by other SCC-nodes is transferred to the IN-nodes.

5. IN: Finally, use Eq. (10) for assigning the network value to the nodes in the IN-subnetwork. In this case the BFS should not consider the SCC-nodes since their value has been already transfer-ed to their first neighbors in the IN. However, it should retrieve the T&T departing from the IN. Again, for the IN-nodes treated in step 4, first assign $v_i \mapsto v^{\text{net}}(i) + v_i$.

Notice that if any part of the bow-tie structure contains additional smaller SCCs, these should be treated first, by applying steps two to four.

This dissection of the network into its bow-tie components also reduces the computational problems. Although we perform a BFS for each node and compute the inverse of the resulting adjacency matrix of the subnetwork as seen in Eq. (10), the smaller sizes of the subnetworks allow faster computations.

To summarize, using one of the three adjacency matrices estimating direct control, $\mathcal{C} \in \{L, T, R\}$, we can compute the corresponding network value for a corporation: v_i^{net}. By deducting the operating revenue, we retrieve the network control: c_i^{net}. Operating revenue is taken for the value of the TNCs (v_i). Fig. S3 shows the distribution of the operating revenue of the TNCs and the resulting network value.

3.3 Proving the BFS Methodology Corrects for Cycles

Here we show that the BFS algorithm presented in the last section yields an equivalent computation proposed in the literature to address the problems of the presence of cycles leading to exaggerated network value.

In [6] the notion of network value was introduced based on ownership which corresponds, in the case of control, to

$$v^{\text{net}} = \tilde{\mathcal{C}} v + v, \tag{11}$$

which in [10] was identified as being problematic. The authors hence introduced a new model which overcomes this problem of exaggerated indirect value in presence of cycles by introducing

$$\hat{\mathcal{C}}_{ij} := \mathcal{C}_{ij} + \sum_{k \neq i} \hat{\mathcal{C}}_{ik} \mathcal{C}_{kj}. \tag{12}$$

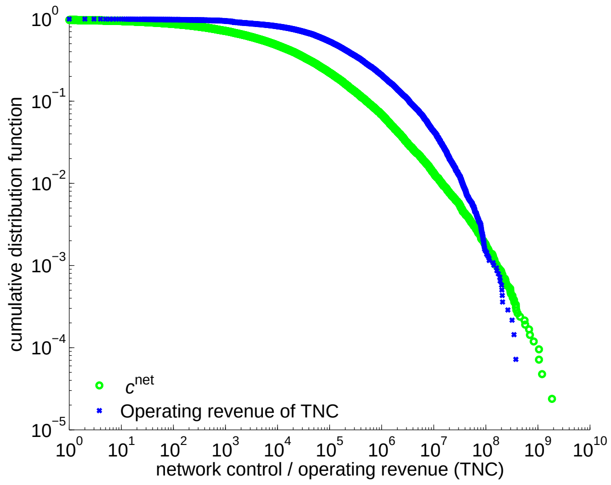

Figure S3. Cumulative distribution function of network control and operating revenue. The network control (TM) in the LCC and the operating revenue of the TNCs in the LCC, from which it is computed, is shown.

This means that the original matrix $\mathcal{C}$ defined in Eq. (3) is corrected by removing all indirect self-loops of any node i. If the network has no cycles, then Eqs. (3) and (12) yield identical solutions.

We introduce here for the first time a correction operator, that incorporates this modification and makes the associated computations clearer

$$\mathcal{D} := \mathrm{diag}((I - \mathcal{C})^{-1})^{-1} = I - \mathrm{diag}(\hat{\mathcal{C}}), \tag{13}$$

where diag(A) is the matrix of the diagonal of the matrix A. It can be shown that

$$\hat{\mathcal{C}} = \mathcal{D}\tilde{\mathcal{C}}. \tag{14}$$

The associated corrected network value can be identified as

$$\hat{v}^{\mathrm{net}} = \mathcal{D}v^{\mathrm{net}} = \hat{\mathcal{C}}v + \mathcal{D}v. \tag{15}$$

Our proposed methodology also corrects for cycles in an equivalent way. This can be seen as follows. By applying the BFS algorithm to node i, we extract the adjacency matrix $B(i)$ of the subnetwork of nodes downstream. From Eq. (12) it holds by construction that

$$\tilde{B}(i)_{ij} = \hat{\mathcal{C}}_{ij} - \hat{\mathcal{C}}_{ii}, \tag{16}$$

where $\tilde{B}(i)$ is defined equivalently to Eq. (2). In a more compact notation

$$\tilde{B}(i)_{i*} = \hat{\mathcal{C}}_{i*} - [\mathrm{diag}(\hat{\mathcal{C}})]_{i*}. \tag{17}$$

Employing Eq. (13) we find that $\tilde{B}(i)_{i*} + I_{i*} = \hat{\mathcal{C}}_{i*} + \mathcal{D}_{i*}$, or equivalently

$$\hat{\mathcal{C}}_{i*}v + \mathcal{D}_{i*}v = \mathcal{D}_{i*}(\tilde{\mathcal{C}}_{i*}v + v_i) = \mathcal{D}_{i*}v^{\mathrm{net}} =: \hat{v}_i^{\mathrm{net}} \tag{18}$$

$$= \tilde{B}_{i*}(i)v + v_i = c^{\mathrm{net}}(i) + v_i =: v^{\mathrm{net}}(i). \tag{19}$$

This concludes that our BFS method and the results in [10] are identical: $\hat{v}_i^{\mathrm{net}} = v^{\mathrm{net}}(i)$.

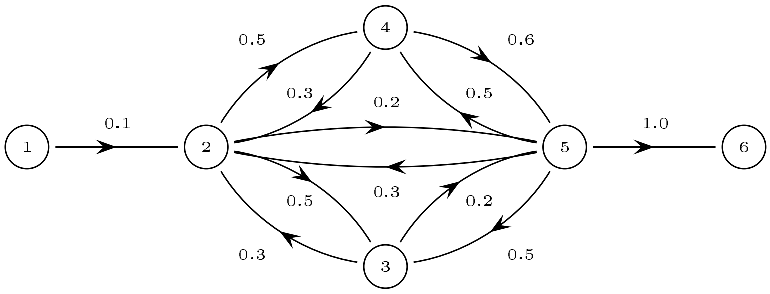

Figure S4. Simple bow-tie network topology. Example with a high degree of interconnectedness of the firms in the strongly connected component (SCC).

3.4 An Illustrated Example

Consider the network illustrated in Figure S4. It is an example of a simple bow-tie network topology. The SCC is constructed in a way to highlight the problem of cross-shareholdings. Hence there are many cycles of indirect ownership originating and ending in each firm in the core of the bow-tie.

We assume the underlying value of each firm to be one, i.e., $v = (1, 1, 1, 1, 1, 1)^t$, where t denotes the transposition operation. Moreover, we will employ the TM, hence $\mathcal{C}_i j = W_{ij}$. This results in the network value and the integrated value to be

$$v^{\text{net}} = \begin{pmatrix} 6 \\ 50 \\ 27 \\ 49 \\ 55 \\ 1 \end{pmatrix}, \tag{20}$$

using Eq. (8).

So although the total value present in the network is $6 = \sum_i v_i$, firm 5 has an disproportionately large network control of $v_5^{\text{net}} = 54$, highlighting the problem of overestimating the control in the presence of cycles.

Employing the corrections proposed in [10], i.e. by computing the correction operator defined in Eq. (13), one finds

$$\mathcal{D} = \begin{pmatrix} 1.000 & 0 & 0 & 0 & 0 & 0 \\ 0 & 0.100 & 0 & 0 & 0 & 0 \\ 0 & 0 & 0.162 & 0 & 0 & 0 \\ 0 & 0 & 0 & 0.095 & 0 & 0 \\ 0 & 0 & 0 & 0 & 0.086 & 0 \\ 0 & 0 & 0 & 0 & 0 & 1.000 \end{pmatrix}. \tag{21}$$

From this, the corrected values can be computed from Eq. (15)

$$\hat{v}^{\text{net}} = \begin{pmatrix} 6.000 \\ 5.000 \\ 4.378 \\ 4.667 \\ 4.714 \\ 1.000 \end{pmatrix}. \tag{22}$$

The correction reduces the values of the firms in the core of the bow-tie by approximately one order of magnitude. This confirms that $\hat{v}^{\text{net}}$ and $\hat{c}^{\text{net}}$ are indeed the right measures to consider in the presence of SCCs in the network.

Unfortunately, this example also highlights the second problem of the methodology. It is clear, that root nodes accumulating all the control. As mentioned, our proposed algorithm remedies this problem while still correcting for the overestimation in cycles. One finds from Eq. (10) that

$$\begin{pmatrix} v^{\text{net}}(1) \\ v^{\text{net}}(2) \\ v^{\text{net}}(3) \\ v^{\text{net}}(4) \\ v^{\text{net}}(5) \\ v^{\text{net}}(6) \end{pmatrix} = \begin{pmatrix} 1.500 \\ 5.000 \\ 4.378 \\ 4.667 \\ 4.714 \\ 1.000 \end{pmatrix}, \tag{23}$$

illustrating the change from $v_1^{\text{net}} = \hat{v}_1^{\text{net}} = 6 \geq v^{\text{net}}(1) = 1.5$.

To summarize, employing v^{net} for the computation of control in networks with bow-tie topology overestimates the level of control in the SCC by construction. Using $\hat{v}^{\text{net}}$ on the other hand always assigns the root nodes the highest control. Only the measure $v^{\text{net}}(.)$ puts root and SCC-nodes on par with each other and the leaf-nodes, allowing for the first time an accurate analysis of the control of each node in the network.

3.5 Relations To Previous Work

To summarize, the relation the existing work is as follows. The notion of network value[3] was introduced in [6], in addition to the integrated ownership matrix. This matrix was later corrected in [10].

The notion of network control was first defined in [5] without any of the corrections described above. Because the networks analysed there comprised only listed companies and their direct shareholders, it was sufficient to apply the uncorrected methodology due to the absence of long indirect paths, see SI Sec. 3. In contrast, in the present work, the full-fledged methodology with all the corrections is required in order to consistently compute the flow of control. This resulted in the introduction of the correction operator and its application to the network value and network control. As mentioned, this allowed us to identify a second problem with the methodology. Subsequently, we have incorporated these insights into an algorithm that is suitable for large networks, correcting all potential problems with computing control. Finally, we also uncover the relationship between network control and network value.

[3]Although the authors only considered the case of ownership and not that of control, their methods are equivalent to the definition of control employing the LM.

4 Degree and Strength Distribution Analysis

The study of the node degree refers to the distribution of the number of in-going and out-going relations. The number of outgoing links of a node corresponds to the number of firms in which a shareholder owns shares. It is a rough measure of the portfolio diversification. The in-degree corresponds to the number of shareholders owning shares in a given firm. It can be thought of as a proxy for control fragmentation. In the TNC network, the out-degree can be approximated by a power law distribution with the exponent -2.15 (see Fig. S5A). The majority of the economic actors points to few others resulting in a low out-degree. At the same time, there are a few nodes with a very high out-degree (the maximum number of companies owned by a single economic actor exceeds 5000 for some financial companies). On the other hand, the in-degree distribution, i.e., the number of shareholders of a company, behaves differently: the frequency of nodes with high in-degree decreases very fast. This is due to the fact that the database cannot provide all the shareholders of a company, especially those that hold only very small shares.

Next to the study of the node degree, we also investigate the strength which is defined as $\sum_j W_{ij}$, that is, the sum of all the weighed participations a company i has in other companies j (see Fig. S5B). It is a measure of the weight connectivity and gives information on how strong the ownership relationships of each node are.

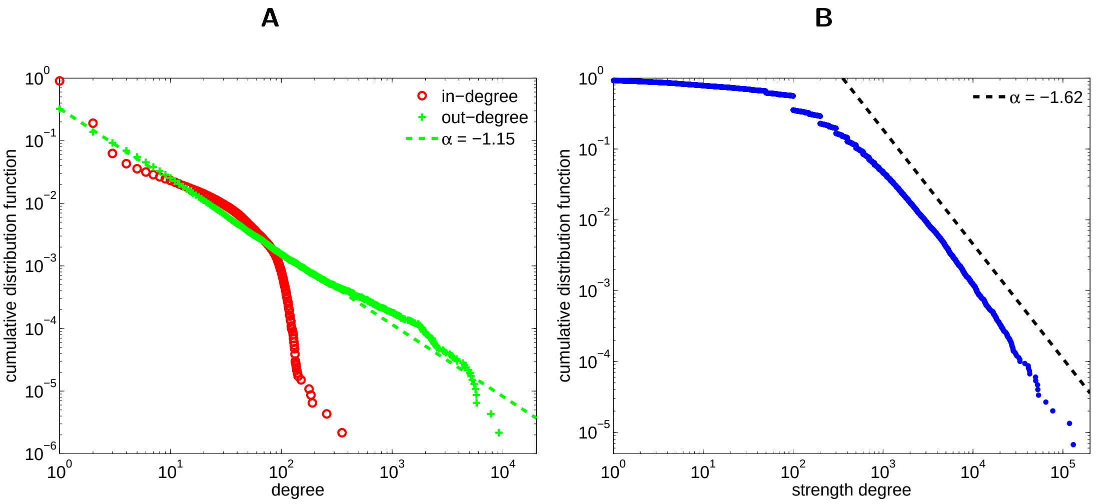

Figure S5. Various distribution functions. (**A**) Cumulative distribution function of the in- and out-degree of the nodes in the LCC (log-log scale). The power-law exponent for the corresponding probability density function of the out-degree is estimated to be -2.15. (**B**) Cumulative distribution function of the node strength in the LCC (log-log scale). As a reference, a power-law with an exponent of -1.62 is displayed.

5 Connected Component Analysis

Ownership relations between companies create formal ties among them. In a strongly connected component (SCC, see SI Sec. 7), all firms reach via an ownership pathway all other firms, thus owning each other indirectly to some extent. In contrast, in a weakly CC firms can reach each other only if one ignores the direction of the ownership links. This is still a situation of interest from an economic point of view because the flow of knowledge and information is not restricted by the direction of the link. The number and the size distribution of the CC provide a measure of the fragmentation of the market. We find that the TNC network consists of 23825 CC. A majority of the nodes (77%) belong to the LCC (largest connected component) with 463006 economic actors and 889601 relations. The remaining nodes belong to CCs with sizes at least 2000 times smaller. The second largest CC contains 230 nodes and 90% of the CC have less than 10 nodes (see Fig. S6).

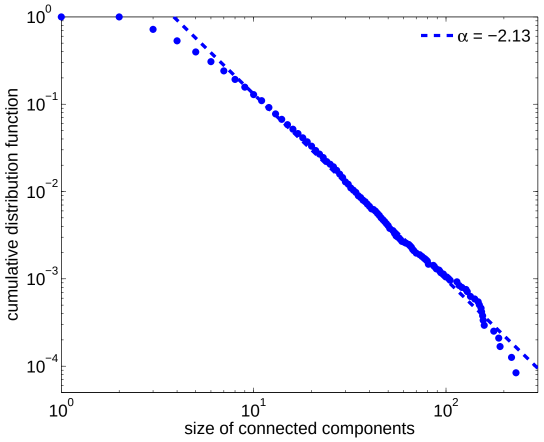

Figure S6. Cumulative distribution function of the size of the connected components. The data point representing the LCC is not shown, as it is three orders of magnitude larger than second largest (with 230 nodes) and completely offset. As a comparison, a power-law with exponent -3.13 ($= \alpha - 1$) is shown.

From a geographical point of view, the LCC includes companies from 191 countries. Of these, 15491 are TNCs (about 36% of all TNCs but accounting for 94.2% of the total operating revenue) from 83 different countries. The firms that are PCs are much more numerous (399696) and are located in only 38 countries. Finally, there are 47819 SHs from 190 countries. This means that shareholders from all around the world hold shares in TNCs located in a more restricted number of countries, which, in turn, further concentrates their ownership shares of PCs in an even smaller number of countries, mainly Europe and the US.

In addition, a sector analysis of the LCC shows that the most represented industries are the business activities sector, with 130587 companies, followed by the services sector with 99839 companies and the manufacturing sector with 66212 companies. On the other hand, surprisingly, the financial intermediaries sector counts only 46632 companies. However, if we distinguish between in-going and out-going relations, the financial intermediaries hold the largest number of shares (341363). Instead, the manufacturing and services sectors, with respectively 182699 and 170397 companies, have the companies with the most shareholders.

6 Bow-Tie Component Sizes

Does a bow-tie structure and the relative size of its IN, OUT and core result from specific economic mechanisms, or could it be explained by a random network formation process? For correlated networks, as in our case, there is no suitable theoretical prediction [11]. Heuristically, one could address the issue by performing a random reshuffling of links. However, this would violate economic constraints. For instance, exchanging a 10% ownership share in a small company with 10% in a big one requires the modification of the budget of the owner. In addition, the procedure is computationally cumbersome for large data sets.

7 Strongly Connected Component Analysis

Cross-shareholdings, or strongly connected components (SCCs) in graph theory, are sub-network structures where companies own each other directly or indirectly through a chain of links (see Fig. S7). Graphically speaking, this means that they form cycles and are all reachable by every other firm in the SCC.

In economics, this kind of ownership relation has raised the attention of different economic institutions, such as the antitrust regulators (which have to guarantee competition in the markets), as well as that of the companies themselves. They can set up cross-shareholdings for coping with possible takeovers, directly sharing information, monitoring and strategies reducing market competition.

In our sample we observe 2219 direct cross-shareholdings (4438 ownership relations), in which 2303 companies are involved and represent 0.44% of all the ownership relations (see Fig. S7A). These direct cross-shareholdings are divided among the different network actors as follow:

- 861 between TNCs;
- 563 between TNCs and PCs;
- 717 between PCs;
- 78 between SHs.

When there is a cross-shareholding involving three companies (see an example in Fig. S7B), many combinations of indirect paths are possible. In our network we observe the following ones:

- 829 of the type: $A \rightarrow B \rightarrow C \rightarrow A$;
- 4.395 of the type: $A \leftrightarrow B \rightarrow C \rightarrow A$;
- 8.963 of the type: $A \leftrightarrow B \leftrightarrow C \rightarrow A$;
- 3.129 of the type: $A \leftrightarrow B \leftrightarrow C \leftrightarrow A$.

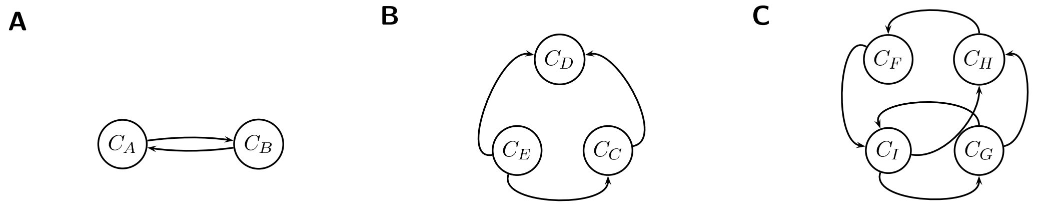

Figure S7. Examples of existing cross-shareholdings. (**A**) Mutual cross-shareholding. (**B**) Possible cross-shareholding with three nodes. (**C**) Cross-shareholding of higher degree.

Next to these simple examples, we also find many SCCs with bigger sizes. Note that smaller SCCs can be embedded in bigger ones. For instance, in the SCC in Fig. S7C there is also one cross-shareholding between the nodes C_I and C_G. In total there are 915 unique SCCs, of which almost all (83.7%) are located in the LCC. Focusing only on the LCC, there is one dominant SCC: it is comprised of 1318 companies in 26 countries. We define the bow-tie structure in the LCC by taking this SCC as its core (in the main text, we only refer to this SCC). The next smallest SCC contains 286 companies. This is a group of Taiwanese firms located in the OUT of the bow-tie. The remaining 99.7% of SCCs in the LCC have sizes between two and 21. The biggest SCC outside the LCC contains 19 firms.

8 Network Control Concentration

8.1 Control of Financial Institutions

One meaning of control in the corporate finance literature is the frequency by which a shareholder is able to influence the firm' strategic decision during the official voting [12]. Differently, in this work, by control we mean how much economic value of companies a shareholder is able to influence. Moreover, we did not limit our focus on the control of a shareholder of a single firm. Instead, we look at the control each shareholder has over its whole portfolio of directly and indirectly owned firms. As a result, the shareholders with a high level of control are those potentially able to impose their decision on many high-value firms. The higher a shareholder's control is, the higher its power to influence the final decision. In this sense, our notion of control can be related to Weber's definition of "power", i.e. the probability of an individual to be able to impose their will despite the opposition of the others [13].

In the literature on corporate control there is a debate on weather financial institutions really exert the control associated with their ownership shares. On the one hand, they are not supposed to seek an active involvement in the companies' strategies. However, some works argue that institutional investors, including banks and mutual funds, do exert control to some extent [14–17]. In particular, the outcome of votes can be influenced by means of informal discussions, in which pro-management votes are used as a bargaining chip (e.g., in exchange of business related "favors" or in negotiating the extension of credit)[4]. On the contrary, [18] and [19] find that mutual funds, which typically hold large blocks of shares, vote against the management (i.e., in favor of corporate governance proposals) only 33% of the times (in the case of Fidelity Fund). However, they do so in more than 60%, on average, in other 11 cases analysed. These results are suggested to originate mainly from a conflict of interest, where the benefits of providing pension plan management to client corporations outweighs the possible benefits gained from increased shareholder value. However, while some mutual funds are reticent to exercise their power during voting mainly in the US, an activist stance is observed for some smaller funds and when operating outside the US [19]. In any case, in our study US mutual funds represent only a small fraction of all global financial institutions. In general, 49 mutual funds, identified by the NACE code 6714, are among the 737 top power-holders (see main text, Sec. Concentration of Control).

8.2 Relation to the Rich Club Phenomenon

The so-called rich club phenomenon [20, 21] refers to the fact that in some complex networks the nodes with the highest degree tend to be connected among each other. Being based solely on node degree, rich club indices are not suitable for ownership networks, in which *indirect* and *weighted* paths matter. Moreover, in order to benchmark the resulting value of rich club indices, it is usually necessary to reshuffle the links in the network. This would be a problem in our network because it would lead to economically unviable ownership networks. Notice, however, that the core of the TNC network could be seen as a generalization of the rich club phenomenon with control in the role of degree. Thus, future work should look into this issue more in depth.

8.3 Top Control-Holders Ranking

This is the first time a ranking of economic actors by global control is presented. Notice that many actors belong to the financial sector (NACE codes starting with 65,66,67) and many of the names are well-known global players. The interest of this ranking is not that it exposes unsuspected powerful players. Instead, it

[4]For example, a mutual fund owning some percent of a large corporation may try to impose job cuts because of a weak economic situation. This can happen: (i) without voting and (ii) although the fund does not plan to keep these shares for many years. In this case, the influence of the mutual fund has a direct impact on the company and its employees. Furthermore, mutual funds with shares in many corporations may try to pursue similar strategies across their entire portfolio.

shows that many of the top actors belong to the core. This means that they do not carry out their business in isolation but, on the contrary, they are tied together in an extremely entangled web of control. This finding is extremely important since there was no prior economic theory or empirical evidence regarding whether and how top players are connected. Finally, it should be noted that governments and natural persons are only featured further down in the list.

Table S1. Top 50 control-holders. Shareholders are ranked by network control (according to the threshold model, TM). Column indicate country, NACE industrial sector code, actor's position in the bow-tie sections, cumulative network control. Notice that NACE code starting with 65,66,67 belong to the financial sector.

Rank	Economic actor name	Country	NACE code	Network position	Cumul. network control (TM, %)
1	BARCLAYS PLC	GB	6512	SCC	4.05
2	CAPITAL GROUP COMPANIES INC, THE	US	6713	IN	6.66
3	FMR CORP	US	6713	IN	8.94
4	AXA	FR	6712	SCC	11.21
5	STATE STREET CORPORATION	US	6713	SCC	13.02
6	JPMORGAN CHASE & CO.	US	6512	SCC	14.55
7	LEGAL & GENERAL GROUP PLC	GB	6603	SCC	16.02
8	VANGUARD GROUP, INC., THE	US	7415	IN	17.25
9	UBS AG	CH	6512	SCC	18.46
10	MERRILL LYNCH & CO., INC.	US	6712	SCC	19.45
11	WELLINGTON MANAGEMENT CO. L.L.P.	US	6713	IN	20.33
12	DEUTSCHE BANK AG	DE	6512	SCC	21.17
13	FRANKLIN RESOURCES, INC.	US	6512	SCC	21.99
14	CREDIT SUISSE GROUP	CH	6512	SCC	22.81
15	WALTON ENTERPRISES LLC	US	2923	T&T	23.56
16	BANK OF NEW YORK MELLON CORP.	US	6512	IN	24.28
17	NATIXIS	FR	6512	SCC	24.98
18	GOLDMAN SACHS GROUP, INC., THE	US	6712	SCC	25.64
19	T. ROWE PRICE GROUP, INC.	US	6713	SCC	26.29
20	LEGG MASON, INC.	US	6712	SCC	26.92
21	MORGAN STANLEY	US	6712	SCC	27.56
22	MITSUBISHI UFJ FINANCIAL GROUP, INC.	JP	6512	SCC	28.16
23	NORTHERN TRUST CORPORATION	US	6512	SCC	28.72
24	SOCIÉTÉ GÉNÉRALE	FR	6512	SCC	29.26
25	BANK OF AMERICA CORPORATION	US	6512	SCC	29.79
26	LLOYDS TSB GROUP PLC	GB	6512	SCC	30.30
27	INVESCO PLC	GB	6523	SCC	30.82
28	ALLIANZ SE	DE	7415	SCC	31.32
29	TIAA	US	6601	IN	32.24
30	OLD MUTUAL PUBLIC LIMITED COMPANY	GB	6601	SCC	32.69
31	AVIVA PLC	GB	6601	SCC	33.14
32	SCHRODERS PLC	GB	6712	SCC	33.57
33	DODGE & COX	US	7415	IN	34.00
34	LEHMAN BROTHERS HOLDINGS, INC.	US	6712	SCC	34.43
35	SUN LIFE FINANCIAL, INC.	CA	6601	SCC	34.82
36	STANDARD LIFE PLC	GB	6601	SCC	35.2
37	CNCE	FR	6512	SCC	35.57
38	NOMURA HOLDINGS, INC.	JP	6512	SCC	35.92
39	THE DEPOSITORY TRUST COMPANY	US	6512	IN	36.28
40	MASSACHUSETTS MUTUAL LIFE INSUR.	US	6601	IN	36.63
41	ING GROEP N.V.	NL	6603	SCC	36.96
42	BRANDES INVESTMENT PARTNERS, L.P.	US	6713	IN	37.29
43	UNICREDITO ITALIANO SPA	IT	6512	SCC	37.61
44	DEPOSIT INSURANCE CORPORATION OF JP	JP	6511	IN	37.93
45	VERENIGING AEGON	NL	6512	IN	38.25
46	BNP PARIBAS	FR	6512	SCC	38.56
47	AFFILIATED MANAGERS GROUP, INC.	US	6713	SCC	38.88
48	RESONA HOLDINGS, INC.	JP	6512	SCC	39.18
49	CAPITAL GROUP INTERNATIONAL, INC.	US	7414	IN	39.48
50	CHINA PETROCHEMICAL GROUP CO.	CN	6511	T&T	39.78

9 Additional Tables

Table S2. Number of top control-holders (TCHs) located in the SCC and being members of the financial sector (FS). Various intersections thereof. The columns refer to the three models of network control and the TM of network value.

	c^{net} (LM, #)	c^{net} (TM, #)	c^{net} (RM, #)	v^{net} (TM, #)
TCH	763	737	648	1791
TCH∩TNC	308	298	259	1241
TCH∩TNC∩SCC	151	147	122	211
TCH∩SCC∩FS	116	115	92	140

Table S3. Concentration of 80% of network control (LM, TM, RM) and network value (TM). The percentages refer to the network controlvalue held by the TCHs according to their location in the SCC and their possible belonging to the FS, and various intersections thereof.

	c^{net} (LM, %)	c^{net} (TM, %)	c^{net} (RM)	v^{net} (TM, %)
TCH∩TNC	54.87	54.63	52.94	63.34
TCH∩TNC∩SCC	39.54	38.37	37.29	30.37
TCH∩SCC∩FS	36.58	35.37	34.90	24.36

Table S4. Probability that a randomly chosen economic actor (TNC or SH) belongs to the group of top control-holders with respect to its position in the network structure. The first column refers to all top control-holders (TCHs), the second column to the first 50 TCH.

	All TCH	First 50 TCH
IN	6.233%	0.273%
SCC	49.831%	11.525%
OUT	0.432%	0%
T&T	0.413%	0.002%
OCC	0.016%	0%

References

1. OECD (2000) *The OECD Guidelins for Multinational Enterprises* (www.oecd.org).
2. Goergen, M, Martynova, M, Renneboog, L (2005) Corporate governance convergence: evidence from takeover regulation reforms in Europe. *Oxford Rev. Econ.Pol.* 21:243–268.
3. The Deminor Group (2005) Application of the one share - one vote principle in europe., (http://www.abi.org.uk/Bookshop/), Technical report.

4. La Porta, R, de Silanes, FL, Shleifer, A (1999) Corporate ownership around the world. *J. Finance* 54:471–517.

5. Glattfelder, JB, Battiston, S (2009) Backbone of complex networks of corporations: The flow of control. *Phys. Rev. E* 80.

6. Brioschi, F, Buzzacchi, L, Colombo, M (1989) Risk capital financing and the separation of ownership and control in business groups. *J. Bank. Financ.* 13:747–772.

7. Bonacich, P (1987) Power and centrality: A family of measures. *Amer. J. Sociol.* pp 1170–1182.

8. Ballester, C, Calvo-Armengol, A, Zenou, Y (2006) Who's who in networks. wanted: the key player. *Econometrica* pp 1403–1417.

9. Hubbell, C (1965) An input-output approach to clique identification. *Sociometry* pp 377–399.

10. Baldone, S, Brioschi, F, Paleari, S (1998) Ownership Measures Among Firms Connected by Cross-Shareholdings and a Further Analogy with Input-Output Theory. *4th JAFEE International Conference on Investment and Derivatives.*

11. Dorogovtsev, S, Mendes, J, Samukhin, A (2001) Giant strongly connected component of directed networks. *Phys. Rev. E* 64:25101.

12. Davis, G (2008) A new finance capitalism? Mutual funds and ownership re-concentration in the United States. *Europ. Manage. Rev.* 5:11–21.

13. Weber, M (1922) Wirtschaft und Gesellschaft, Tübingen. *Max Weber im Kontext. InfoSoftWare Karsten Worm, Berlin* 2.

14. Santos, J, Rumble, A (2006) The American keiretsu and universal banks: Investing, voting and sitting on nonfinancials' corporate boards. *J. Finan. Econ.* 80:419–454.

15. Becht, M, Bolton, P, Röell, A, Roosevelt, A (2005) Corporate governance and control. *NBER.*

16. Gillan, S, Starks, L (2000) Corporate governance proposals and shareholder activism: The role of institutional investors. *J. Finan. Econ.* 57:275–305.

17. Davis, G, Thompson, T (1994) A social movement perspective on corporate control. *Admin. Sci. Quart.* 39:141–173.

18. Davis, G, Kim, E (2007) Business ties and proxy voting by mutual funds. *J. Finan. Econ.* 85:552–570.

19. Davis, G (2008) A new finance capitalism? Mutual funds and ownership re-concentration in the United States. *Euro. Manage. Rev.* 5:11–21.

20. Colizza, V, Flammini, A, Serrano, M, Vespignani, A (2006) Detecting rich-club ordering in complex networks. *Nat. Phy.* 2:110–115.

21. Fagiolo, G, Reyes, J, Schiavo, S (2009) World-trade web: Topological properties, dynamics, and evolution. *Phys. Rev. E* 79:36115.
